# Chronic Coronary Syndrome in Frail Old Population

**DOI:** 10.3390/life12081133

**Published:** 2022-07-27

**Authors:** Adina Carmen Ilie, Sabinne Marie Taranu, Ramona Stefaniu, Ioana Alexandra Sandu, Anca Iuliana Pislaru, Calina Anda Sandu, Ana-Maria Turcu, Ioana Dana Alexa

**Affiliations:** Department of Medical Specialties II, Grigore T. Popa University of Medicine and Pharmacy Iasi, 700115 Iași, Romania; adina.ilie@umfiasi.ro (A.C.I.); ramona.stefaniu@umfiasi.ro (R.S.); ioana0sandu@gmail.com (I.A.S.); pislaru.anca2@gmail.com (A.I.P.); sandu.calina@gmail.com (C.A.S.); ana_turcu2000@yahoo.com (A.-M.T.); ioana.b.alexa@gmail.com (I.D.A.)

**Keywords:** frailty, chronic coronary syndrome, elderly

## Abstract

The demographic trend of aging is associated with an increased prevalence of comorbidities among the elderly. Physical, immunological, emotional and cognitive impairment, in the context of the advanced biological age segment, leads to the maintenance and precipitation of cardiovascular diseases. Thus, more and more data are focused on understanding the pathophysiological mechanisms underlying each fragility phenotype and how they potentiate each other. The implications of inflammation, sarcopenia, vitamin D deficiency and albumin, as dimensions inherent in fragility, in the development and setting of chronic coronary syndromes (CCSs) have proven their patent significance but are still open to research. At the same time, the literature speculates on the interdependent relationship between frailty and CCSs, revealing the role of the first one in the development of the second. In this sense, depression, disabilities, polypharmacy and even cognitive disorders in the elderly with ischemic cardiovascular disease mean a gradual and complex progression of frailty. The battery of tests necessary for the evaluation of the elderly with CCSs requires a permanent update, according to the latest guidelines, but also an individualized approach related to the degree of frailty and the conditions imposed by it. By summation, the knowledge of frailty screening methods, through the use of sensitive and individualized tools, is the foundation of secondary prevention and prognosis in the elderly with CCSs. Moreover, a comprehensive geriatric assessment remains the gold standard of the medical approach of these patients. The management of the frail elderly, with CCSs, brings new challenges, also from the perspective of the treatment particularities. Sometimes the risk–benefit balance is difficult to achieve. Therefore, the holistic, individualized and updated approach of these patients remains a desired objective, by understanding and permanently acquiring knowledge on the complexity of the frailty syndrome.

## 1. Introduction

### 1.1. The Demographic Trend of Aging

The old population continues to increase worldwide, being highly influenced by important regressions in the main causes of mortality. The demographic changes are reflected in society, raising the needs and costs of health care yearly. Worldwide, the proportion of people over the age of 65 is expected to exceed 25% by 2030, and in Europe, the number of older people will increase to 152.6 million in 2060 from 87.5 million in 2010 [1]. At the same time, the growing trend of demographic aging is associated with a high prevalence of coronary artery disease (CAD) in both men and women. The mortality risk and morbidity attributed to chronic coronary syndromes are increased due to aging (especially after 75 years) and due to a high incidence of comorbidities (e.g., hypertension, diabetes, chronic kidney disease, etc.).

With global aging, frailty has become a common condition in the old population. Frailty is highly related to the incidence and unfavorable prognosis of atherosclerotic coronary heart disease [2].

The literature reports two-way associations between cardiovascular disease and frailty. Although peripheral arterial disease and heart failure have been more frequently linked to frailty, current studies focus on broader directions, considering the impact and relationship between chronic coronary syndromes and frailty [3].

### 1.2. Chronic Coronary Syndromes. Definition, Prevalence and Features in Senior Patients

Of all types of cardiovascular diseases, coronary syndrome showed the highest mortality rate, resulting in approximately 659,000 deaths and 805,000 coronary events yearly in the United States [4].

The pathological foundation of coronary heart disease is based on the permanent accumulation of atherosclerotic plaque in the epicardial arteries. It can manifest as obstructive or non-obstructive. Lifestyle regulations, medication and invasive procedures having therapeutic and diagnostic roles can influence the evolution of this process, by stabilizing or regressing the disease. CAD is known by its long periods of stability, but we have to consider the possibility of an acute atherothrombotic event due to plaque rupture or erosion. This event will increase the risk of turning into unstable angina, at any time. Even in apparently silent clinical periods, the disease is usually progressive, and therefore severe. The fact that this pathology implies a dynamic manifestation, we can integrate it in two different clinical presentations known as acute coronary syndromes (ACSs) and chronic coronary syndromes (CCSs) [5,6].

To briefly define chronic coronary syndromes is a challenge, as they are identified by the different evolutionary stages of coronary heart disease, excluding the states in which an acute coronary artery thrombosis dominates the clinical presentation (i.e., ACS) [5,7].

Studies show an increased risk in the old population to develop CCSs. Despite this fact, it seems like this group mostly remains insufficiently diagnosed and treated. The atypical character of the symptoms in a senior patient is recognized, thus delaying the timing of the definite diagnosis [5].

### 1.3. Frailty. Definition, Prevalence and Features in Senior Patients

Frailty is a geriatric syndrome, encompassing different and complex phenotypes, from the physical to the immunological and cognitive components. Frailty is identified by the state of gradual and absolute decline, regardless of the aspects in which it occurs. Geriatric syndromes such as functional dependence, cognitive impairment and malnutrition are in most cases caused by physical frailty which implies decreased muscle strength, a loss of endurance, decreased physiological function and a decreased ability to adapt to stress. Studies have shown clear interactions between frailty status and the complications of coronary heart disease [2,8,9]. Therefore, the evaluation of frailty in seniors has become an indirect way of assessing the true adverse events associated with chronic coronary syndromes.

Fried et al. proposed five dimensions of frailty, which continue to be the diagnostic criteria for this syndrome: decreased muscle strength, decreased walking speed, fatigue, decreased physical activity and involuntary weight loss [2]. Despite its wide use, the Fried phenotype only assesses the physical component of frailty [10]. Other methods of evaluation assess other components, but the golden standard in diagnosing or assessing frailty is the geriatric evaluation.

There are also revealing data confirming a relationship of interdependence and mutual empowerment between CCSs and frailty. Plurivascular disease (40%), tortuosity and calcifications (80–90%) have been seen in patients 80 years of age with a high prevalence of obstructive coronary heart disease (60%) in autopsy studies [6].

However, there is a predominant tendency in current studies to speculate on a one-way relationship between CCSs and frailty, so it seems that cardiovascular damage predisposes to frailty rather than vice versa [8,11].

## 2. Pathogeny, Mechanisms and Associated Factors

### 2.1. Pathophysiology of CCSs and Frailty in Senior Patients

The mechanisms surrounding frailty and cardiac aging require special attention due to their role in senior patients’ morbidity, mortality, quality of life and their need for medical assistance and increased medical costs [12].

Frailty often coexists with heart disease due to coexisting pathophysiological changes, aging and numerous comorbidities, all leading to rapid functional decline and sarcopenia. Higher rates of disability, institutionalization and mortality were recorded in senior patients with both frailty and CCSs [12].

In the pathogenesis of CCSs, varied mechanisms were described in which protein degradation, denervation, atrophy and altered fatty acid oxidation played an important role. Moreover, peroxisome proliferation and decreased protein synthesis, commonly in seniors, activate the gamma coactivator-1 alpha receptor (PGC-1α), whose expression is mitochondrial dysfunction. Thus, sarcopenia represented by homeostenosis of muscle metabolism increases predisposition to chronic coronary syndromes. The altered protein, lipid and glucose metabolism (i.e., insulin resistance) and also endothelial dysfunction, a substrate of cardiovascular disease, are precipitated by an ongoing inflammatory process (increased expression of biomarkers), usually encountered in old age [12].

Nicotinamide adenine dinucleotide phosphate (NADPH) oxidase increases the production of reactive oxygen species (ROS) and stimulates the ubiquitin-proteasome system in skeletal muscle, leading to the development of sarcopenia and increasing the occurrence of chronic coronary syndromes [12,13,14,15].

Elevated levels of C-reactive protein, Interleukin 6 (IL-6) and tumor necrosis factor alpha (TNF-α) are also linked to the loss of skeletal muscle mass. At the same time, TNF-α/nuclear factor-κB (NF-kB) catalyzes the synthesis of ROS in mitochondria, leading to the degradation of muscle proteins, sarcopenia and increasing the risk for ischemic events [12,16,17].

The loss of muscle mass and function also contributes to ischemic heart damage. Therefore, knowledge of the pathogenesis of sarcopenia at the cellular mechanism level is important. With aging, the pathways leading to sarcopenia, involving myostatin, phosphatidylinositol 3-kinase and lysosomal catabolism, are affected. Consequently, autophagy as a mechanism of cell preservation is inactivated and damaged. Its constituents remain undegraded, resulting in the alteration of the quality and quantity of muscle mass, leading, in time, to cardiovascular conditions [12,18,19,20].

The abnormal activation of inflammasome NLRP3, a multiprotein signaling complex found in the cytoplasm of the cell, has also been linked to cardiac inflammation, systolic dysfunction and ventricular remodeling that precipitate the onset of ischemic cardiovascular events [12,21,22,23,24].

Vascular aging is defined by mechanisms involving oxidative stress, mitochondrial dysfunction, genomic instability and epigenetic alterations. Moreover, lipid metabolism, extracellular matrix, coagulation/hemostasis impairment and inflammation are considered to have an important role in vascular aging. Lots of the predisposing factors for atherosclerosis, such as the damage to deoxyribonucleic acid from endogenous or exogenous sources, can lead to improper endothelial function, mediated by reduced nitric oxide synthase (eNOS) activity. They promote impaired vasodilator phenomena, the frailty of blood vessels and the growth of the intimal wall, ultimately leading to an increased risk for coronary heart disease. Theories of aging illustrate the mediator role of special proteins known as sirtuins (e.g., SIRT1) in endothelial function, whose impairment leads to increased reactive oxygen reactive species (ROS). The ROS determine the development of vascular senescence and atherosclerosis [6,25].

In addition, research revealed that dehydroepiandrosterone, fibroblast growth factors (e.g., FGF-23), growth-differentiating factor-15 and plasma-associated lipokaline linked to neutrophil gelatinase have been involved in the progression of cardiovascular disease in the senior patient [6].

### 2.2. Implications of Inflammation in the Pathogenesis of CCSs and Frailty

Inflammation, represented by an impaired immune status and changes in function of immune cell subpopulations, is revealed to be closely related to frailty [26].

A meta-analysis of 32 cross-sectional studies in a group of 23,910 old people showed higher levels of inflammation, indicated by elevated serum levels of CRP and IL-6, in those with frailty and pre-frailty compared to those without frailty. At the same time, a positive link between high serum levels of IL-6, CRP and a loss of muscle mass was highlighted, with a reduction in the grip force, and correlations between inflammation and atherosclerosis were reported simultaneously [2,27].

In CCS patients as well as in frail ones, chronic low-grade inflammation, namely a lifetime exposure to the antigen, angiotensin activation, high body mass index, glucose intolerance and redox instability, are predominantly reported. Aside from conventional inflammatory markers, in CCSs and frailty syndrome, thrombotic markers such as factor VIII and D-Dimers are also elevated [28].

Inflammation is a key factor in both CCSs and frailty. In the first one, we already established the role of the inflammatory syndrome in lipoprotein oxidation and plaque activation. In the second, inflammation is shown to activate a catabolic neurohormonal process that implies the redistribution of amino acids from skeletal muscle to other organ systems. This is a cause of sarcopenia, represented by a decrease in muscle mass, with the inherent modification in muscle metabolism and impairment to adapt to stress factors [28,29].

Intermediate monocytes contribute to the proinflammatory status by releasing cytokines (e.g., TNF-α, IL-1β and IL-6) and reactive oxygen species. Alongside elevated lipoprotein levels, they constitute a possible marker for atherosclerosis and increased CVD risk [26].

IL-6 was found to directly activate muscle catabolism by stimulating the ubiquitin-proteome pathway, annihilating the cytoplasm and nucleoprotein in fibrocytes. In this context, the increase in IL-6 is considered an important predictor of decreased motor capacity, especially in older women. Indirectly, the concentrations of growth hormone (GH) and insulin-like growth factor-1 (IGF-1) have lowered, with decreased protein production and sarcopenia [2].

### 2.3. The Role of Albumin and Vitamin D in the Pathogenesis of CCSs and Frailty

A cohort study by Johansen et al. revealed that for each 1 g/dL increment in serum albumin levels, the frailty grades in senior hemodialysis patients, lowered by 0.4 points, thus suggesting that old patients with increased serum protein concentrations had a decreased rate of frailty [2,30]. These data are sustained by other studies on old, frail and non-frail patients with different comorbidities. Moreover, Dai et al. showed that blood serum albumin concentrations in senior patients with frailty and CCSs were significantly lower than that in pre-frailty and non-frailty patients. Serum albumin, a marker of nutritional status, may be an indicator of frailty in senior patients with chronic coronary syndrome [2].

A 25 (OH) D deficit is a negative prognostic factor in old patients with chronic coronary syndrome. The level of 25 (OH) D can be utilized as a marker to evaluate the gravity of CAD. Recent data have shown its role in predicting frailty [31]. Although results are not very specific, they indicate a link between the genetic mechanism of action of the vitamin D receptor (VDR) and that of cell differentiation and protein production. Thus, when vitamin D is low, adhesion to its receptor decreases simultaneously with muscle mass and strength [2].

**Figure 1:**
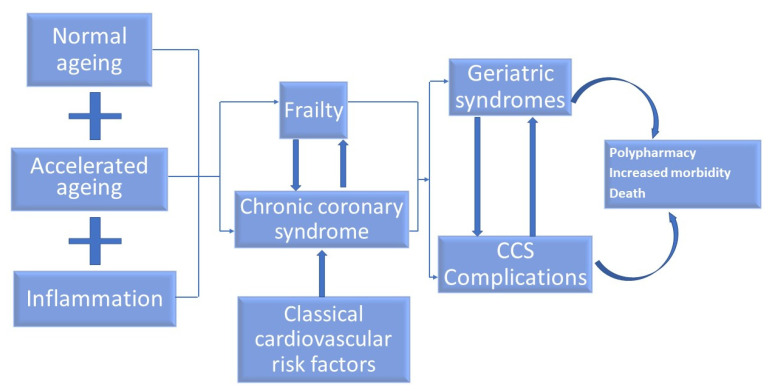
The pathophysiological components between aging, frailty and CCS in old patients. CCS—chronic coronary syndrome.

The pathophysiological components between aging, frailty and CCSs in old patients are synthetized in Figure 1. There are three major factors contributing to both frailty and CCSs in old persons: normal aging, accelerated aging and inflammation. Each one can be an independent factor for frailty and/or CCSs, but they can also be found together as coexisting factors. Normal aging contributes to both frailty and CCSs and the cited mechanisms are: insulin resistance, increased production and accumulation of oxygen reactive species, normal changes in body composition which appear with aging (sarcopenia, central adiposity), homeostatic dysregulation, energy imbalance, neurodegeneration and hormonal dysregulation and inflammageing. Accelerated aging (which includes premature or aging at a rapid pace induced by diseases, genetic disorders or external factors) contributes to frailty and/or CCSs and the involved mechanisms are: increased oxidative stress, telomere shortening, immunosenescence, reduced autophagy and cellular senescence. Inflammation contributes to frailty and/or CCSs and the involved biomarkers are: C-reactive protein, Interleukin 6, tumor necrosis factor alpha (TNF-α) and nuclear factor-κB (NF-kB). All three factors contribute in varied degrees to both frailty and CCSs. Moreover, frailty is well-documented as being an important independent risk factor for CCSs in old patients and CCSs have a negative impact on frailty in old persons. The association of frailty and CCSs has a negative impact on the evolution of geriatric syndromes (especially cognitive impairment, immobility, delirium and iatrogenesis) and the development of CCS complications (acute coronary syndrome, heart failure and arrythmias). All these aspects imply polypharmacy, increased morbidity and death.

## 3. Clinical Presentation and Evaluation of the Frail Senior Patient with CCSs

Diagnosing chronic coronary syndromes in the old aged can be challenging. Senior patients generally have atypical symptoms (e.g., fatigue, dyspnea, nausea, vomiting or postprandial epigastric pain) rather than conventional angina. The presence of hearing or cognitive disorders complicates the anamnesis, and the associated comorbidities delay the diagnosis [6].

Regarding chronic coronary syndromes, the clinical scenarios reported by the latest guidelines are: (i) patients with suspected coronary heart disease and “stable” angina symptoms and/or dyspnea, (ii) patients with a new decompensation of heart failure or left ventricular dysfunction and suspected coronary heart disease, (iii) asymptomatic and symptomatic patients with symptoms stabilized at <1 year after ACS or patients with recent revascularization, (iv) asymptomatic and symptomatic patients > 1 year after initial diagnosis or revascularization, (v) patients with angina pectoris and suspected vasospastic or microvascular angina and (vi) asymptomatic subjects in whom coronary heart disease is detected on screening [5].

All of these are categorized as chronic coronary syndromes but entail various and time-modifiable risks for possible cardiovascular complications. For example, the installation of an ACS can sharply imbalance each of these clinical situations. Risks, in turn, could be precipitated by the improper management of cardiovascular risk factors, inadequate lifestyle adjustments, medical treatment or failure of revascularization. Alternatively, appropriate secondary prevention methods can counterbalance the adverse events of chronic coronary syndromes [5].

Frailty, disability or other processes of the disease that exacerbate fatigue (i.e., pulmonary, skeletal and peripheral artery disease) can veil the perception of the typical symptoms of chronic coronary syndrome by assuming increased oxygen demand for the associated pathologies. Although electrocardiographic anomalies could predict possible negative complications, previous pathologies/abnormalities could limit the usefulness of ECG screening. Transthoracic echocardiography, however, has proven useful in screening for global and segmental ventricular function and structural pathology in old patients [5,6].

There are scarce data about diagnostic and prognostic values of imaging modalities in CCS evaluation in frail old patents compared to the standard population. The few data are for the old and very old population compared to the adult population.

Age influences the validity and reliability of diagnostic tests in CCS patients. In the context of advanced age, exercise stress tests are submaximal undertaken by frail seniors, their diagnostic and prognostic value being less than for young adults. In a study of Newman and Phillips, the sensitivity and specificity of the ECG exercise test in patients aged ≥65 years were 85% and 56%, respectively, and data from a metanalysis, including patients regardless of age, reported that the sensitivity and specificity of ECG exercise testing for the diagnosis of stable CAD were 58% and 62%, respectively [32,33]. Independently of age, bicycle echocardiography is more useful clinically than other tests in the diagnosis of CCSs [32]. Generally, stress testing is better at confirming than infirming CCSs [34]. Exercise echocardiography in old patients reveals resting wall motion abnormalities in 36% of the seniors and new or worsening wall motion abnormalities in 41% of patients. Moreover, in the multivariate analysis, exercise echocardiography variables, such as end systolic volume and ejection fraction, added significantly to clinical factors in predicting mortality [35]. Dobutamine stress echocardiography in old patients generally is well-tolerated, but there is a higher risk for side effects. Asymptomatic hypotension occurred more frequently in the elderly (7% in those <55 vs. 13% in the 55–74 age group vs. 25% in those ≥75). Moreover, any ventricular arrhythmia was more common in the elderly patient population (26% vs. 30% vs. 41%) [36].

Although angiography is frequently used in CCS diagnosis in seniors, many times it is temporized due to frailty and some associated conditions, such as anemic syndromes and chronic kidney disease, the latter coming with high risk of contrast substance nephropathy [37]. Major vascular complications may occur in 3.6% of older patients undergoing diagnostic coronary angiography [6]. When considering cardiac catheterization, the probability of hemorrhagic, embolic and neurological events and acute renal damage induced by the contrast substance should be considered, all of which are increased in geriatric patients. In addition, the old aged with rheumatological or orthopaedical comorbidities may have issues maintaining clinostats and anesthesia. Thus, an individualized approach, comparing the possible advantages and disadvantages of invasive assessment, is required before advising cardiac catheterization to seniors [5,6].

Exploring the presence and rate of ischemia in old patients needs a careful analysis of the usefulness of the diagnosis, as well as the patient’s preferences and objectives of treatment. It is fundamental to establish if the patient in question is eligible for future treatments. A non-invasive diagnostic stress assessment is very important when the subject’s likelihood of developing chronic coronary syndrome is intermediate. Pharmacological stress echocardiography and single-photon emission computed tomography (SPECT), including myocardial perfusion imaging (MPI), have been shown to be effective in stratifying risk in these patients. In those non-eligible for testing, vasoactive agents such as adenosine or dobutamine are needed to cause differential coronary flow or ischemia. This medication associates a high level of iatrogeny, favoring adverse effects such as tachyarrhythmias, hypotension and severe myocardial ischemia. However, depending on the degree of frailty, these agents can be used safely in the old aged [5,6].

The role of magnetic resonance imaging in the evaluation of cardiac ischemia in geriatric patients is still being studied. Stress cardiac magnetic resonance strongly predicted cardiovascular events in people older than 70 years in a cohort of 110 patients. In a cohort of 110 patients who undergo stress CMR, 40.9% of the patients tested positive for ischemia. The median follow-up was 26 months, and in this period, there were 35 cardiovascular events. Therefore, a positive stress CMR with a moderate or severe hypoperfusion defect predicted the appearance of cardiovascular events in the follow-up in patients older than 70 years [38].

Coronary CT angiography is a non-invasive imaging method that can objectify the atherosclerotic rate with no need for pharmacological effort or stress testing. Still, senior people may fail to go through with the test due to their inability to hold their breath. Moreover, they are more likely to acquire atrial fibrillation and renal injury compared to young patients [5,6]. Computed tomography coronary angiography performed in an old population was able to exclude obstructive atheroma in 59% of patients. The positive predictive value of CTCA showing obstructive atheroma was between 74 and 89% when compared with invasive angiography [39].

Regarding the diagnosis of frailty, two main models have been proposed in recent decades. The first is the phenotype model proposed by Fried et al., describing frailty in terms of five physical dimensions: (1) unintentional weight loss of at least 4.5 kg in the last year or ≥5% of body weight in the previous year; (2) self-reported exhaustion identified by two questions from the CES-D depression scale; (3) a reduction in the grip force, adapted according to gender and body mass index (BMI); (4) decreased mobility adjusted to gender and height; and (5) low energy consumption related to physical activity based on the subject’s self-report on the Minnesota Leisure Time Physical Activity Questionnaire (MTLAQ-short version). The lack of any of these items defines patients as “robust”. “Pre-frail” subjects have low scores of one or two, and “frail” patients meet three or more of these criteria [1].

A Canadian study of the frailty index of health and aging (CFI), proposed by Rockwood et al., is based on a cumulative deficit model, comprising 70 items that include clinical signs, symptoms, diseases and comorbidities, in order to construct an individual FI score, compared to the standard one. Various instruments based on these two main models have been used in cardiac rehabilitation [40].

The gold standard for assessing frailty in an old patient remains the Comprehensive Geriatric Assessment (CGA). The CGA consists of a holistic evaluation in different areas of health in seniors, exploring 10 dimensions (cognition, motivation, disability, communication, mobility, balance, bowel/bladder function, nutrition, social ability and comorbidity). The Frailty Index based on a Comprehensive Geriatric Assessment (FI-CGA) is based on the CGA, evaluates the 10 dimensions and classifies patients into three classes of frailty: mild (0–7), moderate (7–13) and severe (>13) [1,41,42].

The different methods in evaluating frailty and its components are described in Table 1 [8].

## 4. Risk Assessment in the Frail Senior Patient with CCS

A frailty assessment using the Clinical Frailty Assessment Scale (CFS) was separately and closely linked to mortality at 6 months of all causes, including after full adjustment for the initial differences in other risk factors [43].

The advanced age segment, increased comorbidities, recurrence of decompensation and prolonged hospitalization (>10 days) and emergency readmission within 30 days of discharge were associated with a higher degree of frailty and, at the same time, with the mortality of any within 30 days of the date of admission. A higher degree of frailty, according to the CFS, is associated with a more reserved prognosis in terms of mortality or the development of complications [43].

Other data suggested a high probability of readmission to hospital and death in frail patients compared to those who are not frail but who are associated with heart failure or chronic coronary syndromes. Polypharmacy and the lack of individualization of treatment are also factors that affect mortality in senior patients with CCSs and explain some of the differences between mortality from any cause and that linked to cardiovascular disease [4].

In the frail old aged, aging accelerates physical and functional decline, which raise the risk of poor health outcomes. The occurrence of frailty is directly proportional to aging and is related to elevated rates of mortality, morbidity, disability and increased health care costs while also being a predictor of poor surgical and interventional outcomes [4]. One meta-analysis evaluating gender differences between frail seniors showed women were considerably more frail, while men had increased mortality. The same data showed that frail old women with CCSs have a higher death rate than frail men of similar age [4,44,45].

During the 6-year follow-up of the NHATS study, it was revealed that seniors with pre-frailty and frailty have higher incidences of all-cause mortality, mainly due to death, stroke and peripheral vascular disease. The means to include frailty evaluation into the care of seniors at risk for CCSs is limited due to low efficacy in the prevention of frailty, no fully efficient therapies of the frailty and limited access to comprehensive geriatric evaluation [46,47,48].

The battery of secondary prevention interventions in frail old people with CCS includes: training, management of risk factors (diet management, smoking abstinence), medical treatment and psychological intervention [1]. Because frailty is a predictor of adverse outcomes in patients with CCSs, both the American Heart Association and the European Society of Cardiology recommend frailty screening in seniors with CAD and the development of intervention plans [5,49].

A holistic evaluation of the high-risk old aged can aid to recognize the factors that determine the development of frailty. It has been shown that aging, limited financial resources, malnutrition and depression are key to the development of frailty in patients with CCSs. The results are significant in the identification of frail adults with CCSs and the emerging need to bring forth preventive interventions [49]. A comprehensive geriatric assessment to recognize frailty is fundamental prior to clinical treatment, as are interventions to limit or reverse frailty in old patients with CCSs [49,50].

## 5. Consequences of the Association of Frailty and CCSs in Senior Patients

The risk for developing CCSs has raised in frail and pre-frail people > 65 years of age. Both frailty and CCSs in the old aged leads to a higher risk of short-term mortality (3 months). As per the frailty phenotype model, the prevalence of CCSs has led to a two- or three-times growth in frail seniors and a tendency to precipitate pre-frailty [1,51].

In hospitalized seniors, malnutrition and hypoalbuminemia may show either a state of frailty or a complication of CCS. The explanation resides in the resemblance and overlap of biological and pathophysiological mechanisms, such as inflammation and age-related subclinical cardiovascular impairment that leads to both conditions [1,52].

In aging patients with CCSs, heart failure (HF) is widespread, and the risk of frailty is 3.4 times bigger, with a high prevalence of pre-frailty (46%) and frailty (40%) The ischemic cardiovascular state predisposes to HF development [53]. Pathophysiologically, it shares common mechanisms with sarcopenia, related especially to inflammation pathways. Thus, it seems that HF can negatively influence gait speed and precipitate malnourishment, resulting in anemia and negative outcomes [1].

The aging of the world’s population is leading to an increase in the incidence and prevalence of geriatric syndromes, such as frailty and disability. The advanced age segment is associated with a series of worsened results related to the link between frailty and CCSs. On these grounds, all other comorbidities, such as sarcopenia, malnutrition, functional dependence, polypharmacy, bleeding risk and thromboembolic events, are precipitated [1,29].

## 6. Treatment Characteristics in the Fragile Senior Patient with CCS

### 6.1. Treatment Goals

The treatment goals in frail old patients, who are associated with CCSs, include increasing life expectancy, minimizing the risk of cardiovascular events, improving or remitting symptoms and returning to normal activities or a good quality of life [11,54].

In senior patients, improving the quality of life, maintaining independence in daily life and controlling symptoms are priorities. In most cases, these issues are more important than prolonging life [55].

### 6.2. Pharmacological Treatment

According to new standards, treatment interventions should take into consideration two ways of approaching frailty. The first one must concentrate on preventing or reducing the progress of frailty while the second will be concerned with the anticipation of side effects in frail seniors. Periodic physical exercise and an adequate diet bring known benefits in the prevention of frailty, but the efficacy of pharmacotherapy is still certain [28].

Concerns have persisted over the past few years that the development of pharmaceutical strategies, such as new lipid-lowering agents in the form of subtilizin/kexin-9 (protein PCSK-9) inhibitors of proprotein convertase, and the use of oral anticoagulants (e.g., Rivaroxaban in patients with increased risk) outshine the role of lifestyle changes. Beta-blockers and calcium channel blockers still are the primary drugs for controlling the symptoms of angina and should be administered depending on the patient’s heart rate, blood pressure and left ventricular function. The utilization of other secondary prevention medications (e.g., angiotensin converting inhibitors and statins) also remains the same [56].

The pharmacological treatment in an old patient must be prescribed considering its particularities: pharmacological and pharmacokinetic changes due to aging, body composition which is different in the old aged compared to adult, pre-existing medication for other conditions, the coexisting comorbidities, the presence of impairments, life expectancy, quality of life, polypharmacy and even the financial aspect [57,58].

Compared to young adults, seniors tend not to adhere to medical regimens, including optimal medical therapy for CCSs. Based on the literature, higher medical treatment compliance improves the survivability rate, even for frail seniors with CCSs [59]. The efficiency of antithrombotic therapy in frail senior patients is comparable to that in young adults, but the first group has a higher risk of bleeding [60].

### 6.3. Interventional Treatment

In the context of worldwide aging and a life expectancy increase, there appeared to be more and more cases of seniors necessitating advanced cardiovascular procedures, such as coronary artery bypass grafting (CABG) and percutaneous coronary intervention (PCI). Increased comorbidity prevalence associated with CCSs predisposes this group to more revascularization complications and poor outcomes. Thus, there is need to preoperatively assess the physiological status of these patients to establish the eligibility for CABG, PCI or pharmacological treatment. In addition to conventional risk stratification, the assessment of frailty is increasingly recognized as an available indicator for predicting outcomes after cardiac surgery [61].

The treatment of chronic coronary syndromes in the old aged suffers from the impasse of vulnerabilities related to both conservative and invasive strategies, such as bleeding, kidney failure and neurological disorders. According to current guidelines, radial access is recommended to be used, when possible, to lower complications at the entry zone when considering an invasive approach for seniors management. It is indicated that diagnostic and revascularization arguments be based on clinical presentation, the rate of ischemia, frailty, life expectancy and other morbidities [5].

Data report that in old patients with isolated CABG, the Preoperative Clinical Frailty Scale (CFS) was a separate predictor of hospital mortality at 30 days. A slow gait has been reported to have an individual predictive role in poor outcomes, even after adjusting for the Thoracic Surgeons Society (STS) score [61]. Debate yet remains as to whether to consider PCI or CABG for senior patients with CCSs and frailty. The presence of asymptomatic myocardial ischemia can be a complication of the frailty status associated with CCSs. Thus, preoperative screening for the frailty of CCS patients remains crucial [61,62].

The frail senior patient with comorbidities has a higher risk of post-CABG mortality compared to young adults [63,64]. Moreover, data show that age over 65, female sex and the presence of comorbidities are associated with higher rates of rehospitalization after CABG [65,66]. CABG should be used cautiously in the frail senior patient.

A single-center cohort study of 3826 patients undergoing cardiac surgery found frailty to be associated with an increased risk of mortality in hospital (adjusted odds ratio, 1.8; 95% CI, 1.1–3.0) and at 2 years (adjusted HR, 1.5; 95% CI, 1.1–2.2) [67]. Another population-based, retrospective, cohort study included 40083 patients undergoing CABG. A total of 8803 (22%) were frail, with a prevalence of frailty higher in the older age groups. Frail patients had higher rates of 30-day mortality than those who were not frail, across all but the ≥85 years age group; at 30 days, 626 patients (1.6%) patients died, of whom 174 (27.8%) were frail. Moreover, at 4 years, there were lower probabilities of survival in patients who were frail at the time of surgery [68]. Frailty has a strong positive relationship with the risk of major adverse cardiac and cerebrovascular events in patients undergoing cardiac surgery; in patients undergoing traditional procedures, such as CABG and/or valve procedures, frailty was associated with an increased OR of mortality, ranging from 1.10 to 2.63 [69].

A PCI is associated with better outcomes in octogenarians compared to young adults, in a higher or at least equal measure. An increased quality of life is one such outcome, but the risks and benefits of the PCI must be considered [70]. However, the efficacy of a PCI is limited by the late addressability of the senior patient, as often they already have complications [60].

A metanalysis which included 2658 seniors identified that the prevalence of frailty ranged from 12.5 to 27.8%. Frailty was associated with increased in-hospital mortality (OR 3.59, 95% CI 2.01–6.42), short-term mortality (OR 6.61, 95% CI 2.89–15.16), as well as long-term mortality (HR 3.24, 95% CI 2.04–5.14) in patients undergoing a PCI. Moreover, prefrailty was a predictor of all-cause mortality in senior patients undergoing a PCI [71]. Frailty also has a negative impact not only on mortality in old patients undergoing a PCI but also in hospital stay and time interval from admission to the PCI. A prospective study including 745 patients undergoing a PCI observed that the time interval from admission to the PCI was longer for frail patients (2.9 ± 5.6 vs. 1.7 ± 3.1 days, *p* < 0.001). After the PCI, frail patients remained in hospital substantially longer than non-frail patients (14.1 ± 26.7 vs. 3.5 ± 8.8 days, *p* < 0.001), and frail patients were nearly five times more likely to die within 30 days after the PCI, compared with non-frail patients (HR 4.8, 95% CI 1.4 to 16.3, *p* = 0.01) [72].

There are scarce data regarding a comparison of an OMT, PCI and CABG in frail old patients. The few data are for old patients with coronary chronic total occlusion, regardless of their frailty status. In these patients, a complete revascularization is more frequently achieved with CABG rather than a PCI, quality of life could be elevated by CABG and the 30-day mortality and 1-year survival were similar between CABG and PCI. However, the authors stated that when patients have a higher surgical risk such as frailty, serious comorbidity or dementia, a PCI seems to be more appropriate [73]. The European Society of Cardiology recommends to adopt a conservative, a noninvasive or a less invasive strategy in frail old patients [72,74].

### 6.4. Monitoring and Rehabilitation Programs

A relationship of interdependence and mutual potentiation has been established between frailty and cardiovascular impairment. Therefore, it is plausible that some rehabilitation measures to reduce frailty, including resistance training or nutritional approach, which involve an increased intake of protein and amino acids, may benefit seniors with CVD and frailty [61].

Although there is much data regarding cardiac rehabilitation and its role in restoring functional capacity and the quality of life in the frail old aged, there are few data assessing the role of cardiac rehabilitation, by improving frailty, in improving the prognosis in patients with CVD, including CCSs. We also interpret this aspect based on the fact that resistance training is not advised in most patients with CVD, due to the fact that it raises the post-cardiac load [61,75].

Most frail old patients eligible for cardiac rehabilitation programs have limitations in accessing them: there is a need for resources in the use of mobile technologies and in the correct performance of physical therapy exercises. However, it is certain that these interventions, and especially those requiring high-intensity training, have not been fully explored in the senior population.

Telemedicine has also been shown to be an additional tool for frail seniors who are unable to participate in cardiovascular rehabilitation exercises in hospitals. New data and reviews have recently showed the advantages of hybrid programs, which use remote monitoring and telerehabilitation platforms. In old patients with HF, the improvement in distance after following these rehabilitation programs for 12 weeks was statistically significant [1,76,77].

Although no significant differences were identified in terms of increased quality of life, mortality and improved functional capacity between conventional and hybrid cardiac rehabilitation programs, greater compliance was shown with those who joined the latter.

Frail seniors with ACSs or CCSs are often excluded from multidisciplinary cardiac rehabilitation measures or programs based on exercise. Future studies will focus on identifying the best frailty assessment tool for developing individualized care models. Cardiac rehabilitation programs at home and telerehabilitation models imagined specifically for frail seniors are enthusiastically advised [1].

Cognitive-behavioral and psychological interventions, exercise-based cardiac rehabilitation, annual influenza vaccination and the holistic assessment of senior patients are the levers of an individualized therapeutic approach, with the chance to reduce disabilities, increase quality of life and increase life expectancy.

## Figures and Tables

**Table 1:** Diagnostic methods of frailty and frailty subtype.

Diagnostic Methods of Frailty	Frailty Subtype				
	Physical	Cognitive	Clinical	Psychological	Social
Fried criteria	x	-	x	-	-
FI-CGA	x	x	x	x	x
CFS	x	-	x	-	-
CSHA-FI	x	x	x	x	-
EFS	x	x	x	-	x
CGA	x	x	x	x	x

FI-CGA = Frailty Index based on a Comprehensive Geriatric Assessment; CFS = Clinical Frailty Scale; CSHA-FI = Canadian Study of Health and Aging Frailty Index; EFS = Edmonton Frailty scale; CGA = Comprehensive Geriatric Evaluation.
